# A Deep Neural Network for Simultaneous Estimation of b Jet Energy and Resolution

**DOI:** 10.1007/s41781-020-00041-z

**Published:** 2020-10-30

**Authors:** A. M. Sirunyan, A. Tumasyan, W. Adam, F. Ambrogi, T. Bergauer, M. Dragicevic, J. Erö, A. Escalante Del Valle, M. Flechl, R. Frühwirth, M. Jeitler, N. Krammer, I. Krätschmer, D. Liko, T. Madlener, I. Mikulec, N. Rad, J. Schieck, R. Schöfbeck, M. Spanring, D. Spitzbart, W. Waltenberger, C.-E. Wulz, M. Zarucki, V. Drugakov, V. Mossolov, J. Suarez Gonzalez, M. R. Darwish, E. A. De Wolf, D. Di Croce, X. Janssen, A. Lelek, M. Pieters, H. Rejeb Sfar, H. Van Haevermaet, P. Van Mechelen, S. Van Putte, N. Van Remortel, F. Blekman, E. S. Bols, S. S. Chhibra, J. D’Hondt, J. De Clercq, D. Lontkovskyi, S. Lowette, I. Marchesini, S. Moortgat, Q. Python, K. Skovpen, S. Tavernier, W. Van Doninck, P. Van Mulders, D. Beghin, B. Bilin, B. Clerbaux, G. De Lentdecker, H. Delannoy, B. Dorney, L. Favart, A. Grebenyuk, A. K. Kalsi, A. Popov, N. Postiau, E. Starling, L. Thomas, C. Vander Velde, P. Vanlaer, D. Vannerom, T. Cornelis, D. Dobur, I. Khvastunov, M. Niedziela, C. Roskas, M. Tytgat, W. Verbeke, B. Vermassen, M. Vit, O. Bondu, G. Bruno, C. Caputo, P. David, C. Delaere, M. Delcourt, A. Giammanco, V. Lemaitre, J. Prisciandaro, A. Saggio, M. Vidal Marono, P. Vischia, J. Zobec, F. L. Alves, G. A. Alves, G. Correia Silva, C. Hensel, A. Moraes, P. Rebello Teles, E. Belchior Batista Das Chagas, W. Carvalho, J. Chinellato, E. Coelho, E. M. Da Costa, G. G. Da Silveira, D. De Jesus Damiao, C. De Oliveira Martins, S. Fonseca De Souza, L. M. Huertas Guativa, H. Malbouisson, J. Martins, D. Matos Figueiredo, M. Medina Jaime, M. Melo De Almeida, C. Mora Herrera, L. Mundim, H. Nogima, W. L. Prado Da Silva, L. J. Sanchez Rosas, A. Santoro, A. Sznajder, M. Thiel, E. J. Tonelli Manganote, F. Torres Da Silva De Araujo, A. Vilela Pereira, C. A. Bernardes, L. Calligaris, T. R. Fernandez Perez Tomei, E. M. Gregores, D. S. Lemos, P. G. Mercadante, S. F. Novaes, SandraS. Padula, A. Aleksandrov, G. Antchev, R. Hadjiiska, P. Iaydjiev, M. Misheva, M. Rodozov, M. Shopova, G. Sultanov, M. Bonchev, A. Dimitrov, T. Ivanov, L. Litov, B. Pavlov, P. Petkov, W. Fang, X. Gao, L. Yuan, M. Ahmad, Z. Hu, Y. Wang, G. M. Chen, H. S. Chen, M. Chen, C. H. Jiang, D. Leggat, H. Liao, Z. Liu, A. Spiezia, J. Tao, E. Yazgan, H. Zhang, S. Zhang, J. Zhao, A. Agapitos, Y. Ban, G. Chen, A. Levin, J. Li, L. Li, Q. Li, Y. Mao, S. J. Qian, D. Wang, Q. Wang, M. Xiao, C. Avila, A. Cabrera, C. Florez, C. F. González Hernández, M. A. Segura Delgado, J. Mejia Guisao, J. D. Ruiz Alvarez, C. A. Salazar González, N. Vanegas Arbelaez, D. Giljanović, N. Godinovic, D. Lelas, I. Puljak, T. Sculac, Z. Antunovic, M. Kovac, V. Brigljevic, D. Ferencek, K. Kadija, B. Mesic, M. Roguljic, A. Starodumov, T. Susa, M. W. Ather, A. Attikis, E. Erodotou, A. Ioannou, M. Kolosova, S. Konstantinou, G. Mavromanolakis, J. Mousa, C. Nicolaou, F. Ptochos, P. A. Razis, H. Rykaczewski, D. Tsiakkouri, M. Finger, M. Finger, A. Kveton, J. Tomsa, E. Ayala, E. Carrera Jarrin, H. Abdalla, S. Elgammal, S. Bhowmik, A. Carvalho Antunes De Oliveira, R. K. Dewanjee, K. Ehataht, M. Kadastik, M. Raidal, C. Veelken, P. Eerola, L. Forthomme, H. Kirschenmann, K. Osterberg, M. Voutilainen, F. Garcia, J. Havukainen, J. K. Heikkilä, V. Karimäki, M. S. Kim, R. Kinnunen, T. Lampén, K. Lassila-Perini, S. Laurila, S. Lehti, T. Lindén, P. Luukka, T. Mäenpää, H. Siikonen, E. Tuominen, J. Tuominiemi, T. Tuuva, M. Besancon, F. Couderc, M. Dejardin, D. Denegri, B. Fabbro, J. L. Faure, F. Ferri, S. Ganjour, A. Givernaud, P. Gras, G. Hamel de Monchenault, P. Jarry, C. Leloup, B. Lenzi, E. Locci, J. Malcles, J. Rander, A. Rosowsky, M.Ö. Sahin, A. Savoy-Navarro, M. Titov, G. B. Yu, S. Ahuja, C. Amendola, F. Beaudette, P. Busson, C. Charlot, B. Diab, G. Falmagne, R. Granier de Cassagnac, I. Kucher, A. Lobanov, C. Martin Perez, M. Nguyen, C. Ochando, P. Paganini, J. Rembser, R. Salerno, J. B. Sauvan, Y. Sirois, A. Zabi, A. Zghiche, J.-L. Agram, J. Andrea, D. Bloch, G. Bourgatte, J.-M. Brom, E. C. Chabert, C. Collard, E. Conte, J.-C. Fontaine, D. Gelé, U. Goerlach, M. Jansová, A.-C. Le Bihan, N. Tonon, P. Van Hove, S. Gadrat, S. Beauceron, C. Bernet, G. Boudoul, C. Camen, A. Carle, N. Chanon, R. Chierici, D. Contardo, P. Depasse, H. El Mamouni, J. Fay, S. Gascon, M. Gouzevitch, B. Ille, Sa. Jain, F. Lagarde, I. B. Laktineh, H. Lattaud, A. Lesauvage, M. Lethuillier, L. Mirabito, S. Perries, V. Sordini, L. Torterotot, G. Touquet, M. Vander Donckt, S. Viret, A. Khvedelidze, Z. Tsamalaidze, C. Autermann, L. Feld, K. Klein, M. Lipinski, D. Meuser, A. Pauls, M. Preuten, M. P. Rauch, J. Schulz, M. Teroerde, B. Wittmer, M. Erdmann, B. Fischer, S. Ghosh, T. Hebbeker, K. Hoepfner, H. Keller, L. Mastrolorenzo, M. Merschmeyer, A. Meyer, P. Millet, G. Mocellin, S. Mondal, S. Mukherjee, D. Noll, A. Novak, T. Pook, A. Pozdnyakov, T. Quast, M. Radziej, Y. Rath, H. Reithler, J. Roemer, A. Schmidt, S. C. Schuler, A. Sharma, S. Wiedenbeck, S. Zaleski, G. Flügge, W. Haj Ahmad, O. Hlushchenko, T. Kress, T. Müller, A. Nowack, C. Pistone, O. Pooth, D. Roy, H. Sert, A. Stahl, M. Aldaya Martin, P. Asmuss, I. Babounikau, H. Bakhshiansohi, K. Beernaert, O. Behnke, A. Bermúdez Martínez, D. Bertsche, A. A. Bin Anuar, K. Borras, V. Botta, A. Campbell, A. Cardini, P. Connor, S. Consuegra Rodríguez, C. Contreras-Campana, V. Danilov, A. De Wit, M. M. Defranchis, C. Diez Pardos, D. Domínguez Damiani, G. Eckerlin, D. Eckstein, T. Eichhorn, A. Elwood, E. Eren, E. Gallo, A. Geiser, A. Grohsjean, M. Guthoff, M. Haranko, A. Harb, A. Jafari, N. Z. Jomhari, H. Jung, A. Kasem, M. Kasemann, H. Kaveh, J. Keaveney, C. Kleinwort, J. Knolle, D. Krücker, W. Lange, T. Lenz, J. Lidrych, K. Lipka, W. Lohmann, R. Mankel, I.-A. Melzer-Pellmann, A. B. Meyer, M. Meyer, M. Missiroli, J. Mnich, A. Mussgiller, V. Myronenko, D. Pérez Adán, S. K. Pflitsch, D. Pitzl, A. Raspereza, A. Saibel, M. Savitskyi, V. Scheurer, P. Schütze, C. Schwanenberger, R. Shevchenko, A. Singh, H. Tholen, O. Turkot, A. Vagnerini, M. Van De Klundert, R. Walsh, Y. Wen, K. Wichmann, C. Wissing, O. Zenaiev, R. Zlebcik, R. Aggleton, S. Bein, L. Benato, A. Benecke, V. Blobel, T. Dreyer, A. Ebrahimi, F. Feindt, A. Fröhlich, C. Garbers, E. Garutti, D. Gonzalez, P. Gunnellini, J. Haller, A. Hinzmann, A. Karavdina, G. Kasieczka, R. Klanner, R. Kogler, N. Kovalchuk, S. Kurz, V. Kutzner, J. Lange, T. Lange, A. Malara, J. Multhaup, C. E. N. Niemeyer, A. Perieanu, A. Reimers, O. Rieger, C. Scharf, P. Schleper, S. Schumann, J. Schwandt, J. Sonneveld, H. Stadie, G. Steinbrück, F. M. Stober, B. Vormwald, I. Zoi, M. Akbiyik, C. Barth, M. Baselga, S. Baur, T. Berger, E. Butz, R. Caspart, T. Chwalek, W. De Boer, A. Dierlamm, K. El Morabit, N. Faltermann, M. Giffels, P. Goldenzweig, A. Gottmann, M. A. Harrendorf, F. Hartmann, U. Husemann, S. Kudella, S. Mitra, M. U. Mozer, D. Müller, Th. Müller, M. Musich, A. Nürnberg, G. Quast, K. Rabbertz, M. Schröder, I. Shvetsov, H. J. Simonis, R. Ulrich, M. Wassmer, M. Weber, C. Wöhrmann, R. Wolf, G. Anagnostou, P. Asenov, G. Daskalakis, T. Geralis, A. Kyriakis, D. Loukas, G. Paspalaki, M. Diamantopoulou, G. Karathanasis, P. Kontaxakis, A. Manousakis-katsikakis, A. Panagiotou, I. Papavergou, N. Saoulidou, A. Stakia, K. Theofilatos, K. Vellidis, E. Vourliotis, G. Bakas, K. Kousouris, I. Papakrivopoulos, G. Tsipolitis, I. Evangelou, C. Foudas, P. Gianneios, P. Katsoulis, P. Kokkas, S. Mallios, K. Manitara, N. Manthos, I. Papadopoulos, J. Strologas, F. A. Triantis, D. Tsitsonis, M. Bartók, R. Chudasama, M. Csanad, P. Major, K. Mandal, A. Mehta, M. I. Nagy, G. Pasztor, O. Surányi, G. I. Veres, G. Bencze, C. Hajdu, D. Horvath, F. Sikler, T.Á. Vámi, V. Veszpremi, G. Vesztergombi, N. Beni, S. Czellar, J. Karancsi, J. Molnar, Z. Szillasi, P. Raics, D. Teyssier, Z. L. Trocsanyi, B. Ujvari, T. Csorgo, W. J. Metzger, F. Nemes, T. Novak, S. Choudhury, J. R. Komaragiri, P. C. Tiwari, S. Bahinipati, C. Kar, G. Kole, P. Mal, V. K. Muraleedharan Nair Bindhu, A. Nayak, D. K. Sahoo, S. K. Swain, S. Bansal, S. B. Beri, V. Bhatnagar, S. Chauhan, R. Chawla, N. Dhingra, R. Gupta, A. Kaur, M. Kaur, S. Kaur, P. Kumari, M. Lohan, M. Meena, K. Sandeep, S. Sharma, J. B. Singh, A. K. Virdi, A. Bhardwaj, B. C. Choudhary, R. B. Garg, M. Gola, S. Keshri, Ashok Kumar, M. Naimuddin, P. Priyanka, K. Ranjan, Aashaq Shah, R. Sharma, R. Bhardwaj, M. Bharti, R. Bhattacharya, S. Bhattacharya, U. Bhawandeep, D. Bhowmik, S. Dutta, S. Ghosh, B. Gomber, M. Maity, K. Mondal, S. Nandan, A. Purohit, P. K. Rout, G. Saha, S. Sarkar, T. Sarkar, M. Sharan, B. Singh, S. Thakur, P. K. Behera, P. Kalbhor, A. Muhammad, P. R. Pujahari, A. Sharma, A. K. Sikdar, D. Dutta, V. Jha, V. Kumar, D. K. Mishra, P. K. Netrakanti, L. M. Pant, P. Shukla, T. Aziz, M. A. Bhat, S. Dugad, G. B. Mohanty, N. Sur, RavindraKumar Verma, S. Banerjee, S. Bhattacharya, S. Chatterjee, P. Das, M. Guchait, S. Karmakar, S. Kumar, G. Majumder, K. Mazumdar, N. Sahoo, S. Sawant, S. Dube, B. Kansal, A. Kapoor, K. Kothekar, S. Pandey, A. Rane, A. Rastogi, S. Sharma, S. Chenarani, E. Eskandari Tadavani, S. M. Etesami, M. Khakzad, M. Mohammadi Najafabadi, M. Naseri, F. Rezaei Hosseinabadi, M. Felcini, M. Grunewald, M. Abbrescia, R. Aly, C. Calabria, A. Colaleo, D. Creanza, L. Cristella, N. De Filippis, M. De Palma, A. Di Florio, W. Elmetenawee, L. Fiore, A. Gelmi, G. Iaselli, M. Ince, S. Lezki, G. Maggi, M. Maggi, J. A. Merlin, G. Miniello, S. My, S. Nuzzo, A. Pompili, G. Pugliese, R. Radogna, A. Ranieri, G. Selvaggi, L. Silvestris, F. M. Simone, R. Venditti, P. Verwilligen, G. Abbiendi, C. Battilana, D. Bonacorsi, L. Borgonovi, S. Braibant-Giacomelli, R. Campanini, P. Capiluppi, A. Castro, F. R. Cavallo, C. Ciocca, G. Codispoti, M. Cuffiani, G. M. Dallavalle, F. Fabbri, A. Fanfani, E. Fontanesi, P. Giacomelli, C. Grandi, L. Guiducci, F. Iemmi, S. Lo Meo, S. Marcellini, G. Masetti, F. L. Navarria, A. Perrotta, F. Primavera, A. M. Rossi, T. Rovelli, G. P. Siroli, N. Tosi, S. Albergo, S. Costa, A. Di Mattia, R. Potenza, A. Tricomi, C. Tuve, G. Barbagli, A. Cassese, R. Ceccarelli, V. Ciulli, C. Civinini, R. D’Alessandro, F. Fiori, E. Focardi, G. Latino, P. Lenzi, M. Meschini, S. Paoletti, G. Sguazzoni, L. Viliani, L. Benussi, S. Bianco, D. Piccolo, M. Bozzo, F. Ferro, R. Mulargia, E. Robutti, S. Tosi, A. Benaglia, A. Beschi, F. Brivio, V. Ciriolo, M. E. Dinardo, P. Dini, S. Gennai, A. Ghezzi, P. Govoni, L. Guzzi, M. Malberti, S. Malvezzi, D. Menasce, F. Monti, L. Moroni, M. Paganoni, D. Pedrini, S. Ragazzi, T. Tabarelli de Fatis, D. Zuolo, S. Buontempo, N. Cavallo, A. De Iorio, A. Di Crescenzo, F. Fabozzi, F. Fienga, G. Galati, A. O. M. Iorio, L. Lista, S. Meola, P. Paolucci, B. Rossi, C. Sciacca, E. Voevodina, P. Azzi, N. Bacchetta, D. Bisello, A. Boletti, A. Bragagnolo, R. Carlin, P. Checchia, P. De Castro Manzano, T. Dorigo, U. Dosselli, F. Gasparini, U. Gasparini, A. Gozzelino, S. Y. Hoh, P. Lujan, M. Margoni, A. T. Meneguzzo, J. Pazzini, M. Presilla, P. Ronchese, R. Rossin, F. Simonetto, A. Tiko, M. Tosi, M. Zanetti, P. Zotto, G. Zumerle, A. Braghieri, D. Fiorina, P. Montagna, S. P. Ratti, V. Re, M. Ressegotti, C. Riccardi, P. Salvini, I. Vai, P. Vitulo, M. Biasini, G. M. Bilei, D. Ciangottini, L. Fanò, P. Lariccia, R. Leonardi, E. Manoni, G. Mantovani, V. Mariani, M. Menichelli, A. Rossi, A. Santocchia, D. Spiga, K. Androsov, P. Azzurri, G. Bagliesi, V. Bertacchi, L. Bianchini, T. Boccali, R. Castaldi, M. A. Ciocci, R. Dell’Orso, S. Donato, G. Fedi, L. Giannini, A. Giassi, M. T. Grippo, F. Ligabue, E. Manca, G. Mandorli, A. Messineo, F. Palla, A. Rizzi, G. Rolandi, S. Roy Chowdhury, A. Scribano, P. Spagnolo, R. Tenchini, G. Tonelli, N. Turini, A. Venturi, P. G. Verdini, F. Cavallari, M. Cipriani, D. Del Re, E. Di Marco, M. Diemoz, E. Longo, P. Meridiani, G. Organtini, F. Pandolfi, R. Paramatti, C. Quaranta, S. Rahatlou, C. Rovelli, F. Santanastasio, L. Soffi, N. Amapane, R. Arcidiacono, S. Argiro, M. Arneodo, N. Bartosik, R. Bellan, A. Bellora, C. Biino, A. Cappati, N. Cartiglia, S. Cometti, M. Costa, R. Covarelli, N. Demaria, B. Kiani, F. Legger, C. Mariotti, S. Maselli, E. Migliore, V. Monaco, E. Monteil, M. Monteno, M. M. Obertino, G. Ortona, L. Pacher, N. Pastrone, M. Pelliccioni, G. L. Pinna Angioni, A. Romero, M. Ruspa, R. Salvatico, V. Sola, A. Solano, D. Soldi, A. Staiano, D. Trocino, S. Belforte, V. Candelise, M. Casarsa, F. Cossutti, A. Da Rold, G. Della Ricca, F. Vazzoler, A. Zanetti, B. Kim, D. H. Kim, G. N. Kim, J. Lee, S. W. Lee, C. S. Moon, Y. D. Oh, S. I. Pak, S. Sekmen, D. C. Son, Y. C. Yang, H. Kim, D. H. Moon, G. Oh, B. Francois, T. J. Kim, J. Park, S. Cho, S. Choi, Y. Go, S. Ha, B. Hong, K. Lee, K. S. Lee, J. Lim, J. Park, S. K. Park, Y. Roh, J. Yoo, J. Goh, H. S. Kim, J. Almond, J. H. Bhyun, J. Choi, S. Jeon, J. Kim, J. S. Kim, H. Lee, K. Lee, S. Lee, K. Nam, M. Oh, S. B. Oh, B. C. Radburn-Smith, U. K. Yang, H. D. Yoo, I. Yoon, D. Jeon, J. H. Kim, J. S. H. Lee, I. C. Park, I. J. Watson, Y. Choi, C. Hwang, Y. Jeong, J. Lee, Y. Lee, I. Yu, V. Veckalns, V. Dudenas, A. Juodagalvis, A. Rinkevicius, G. Tamulaitis, J. Vaitkus, Z. A. Ibrahim, F. Mohamad Idris, W. A. T. Wan Abdullah, M. N. Yusli, Z. Zolkapli, J. F. Benitez, A. Castaneda Hernandez, J. A. Murillo Quijada, L. Valencia Palomo, H. Castilla-Valdez, E. De La Cruz-Burelo, I. Heredia-De La Cruz, R. Lopez-Fernandez, A. Sanchez-Hernandez, S. Carrillo Moreno, C. Oropeza Barrera, M. Ramirez-Garcia, F. Vazquez Valencia, J. Eysermans, I. Pedraza, H. A. Salazar Ibarguen, C. Uribe Estrada, A. Morelos Pineda, J. Mijuskovic, N. Raicevic, D. Krofcheck, S. Bheesette, P. H. Butler, A. Ahmad, M. Ahmad, Q. Hassan, H. R. Hoorani, W. A. Khan, M. A. Shah, M. Shoaib, M. Waqas, V. Avati, L. Grzanka, M. Malawski, H. Bialkowska, M. Bluj, B. Boimska, M. Górski, M. Kazana, M. Szleper, P. Zalewski, K. Bunkowski, A. Byszuk, K. Doroba, A. Kalinowski, M. Konecki, J. Krolikowski, M. Olszewski, M. Walczak, M. Araujo, P. Bargassa, D. Bastos, A. Di Francesco, P. Faccioli, B. Galinhas, M. Gallinaro, J. Hollar, N. Leonardo, T. Niknejad, J. Seixas, K. Shchelina, G. Strong, O. Toldaiev, J. Varela, S. Afanasiev, P. Bunin, M. Gavrilenko, I. Golutvin, I. Gorbunov, A. Kamenev, V. Karjavine, A. Lanev, A. Malakhov, V. Matveev, P. Moisenz, V. Palichik, V. Perelygin, M. Savina, S. Shmatov, S. Shulha, N. Skatchkov, V. Smirnov, N. Voytishin, A. Zarubin, L. Chtchipounov, V. Golovtcov, Y. Ivanov, V. Kim, E. Kuznetsova, P. Levchenko, V. Murzin, V. Oreshkin, I. Smirnov, D. Sosnov, V. Sulimov, L. Uvarov, A. Vorobyev, Yu. Andreev, A. Dermenev, S. Gninenko, N. Golubev, A. Karneyeu, M. Kirsanov, N. Krasnikov, A. Pashenkov, D. Tlisov, A. Toropin, V. Epshteyn, V. Gavrilov, N. Lychkovskaya, A. Nikitenko, V. Popov, I. Pozdnyakov, G. Safronov, A. Spiridonov, A. Stepennov, M. Toms, E. Vlasov, A. Zhokin, T. Aushev, M. Chadeeva, P. Parygin, D. Philippov, E. Popova, V. Rusinov, V. Andreev, M. Azarkin, I. Dremin, M. Kirakosyan, A. Terkulov, A. Baskakov, A. Belyaev, E. Boos, M. Dubinin, L. Dudko, A. Ershov, A. Gribushin, V. Klyukhin, O. Kodolova, I. Lokhtin, S. Obraztsov, S. Petrushanko, V. Savrin, A. Barnyakov, V. Blinov, T. Dimova, L. Kardapoltsev, Y. Skovpen, I. Azhgirey, I. Bayshev, S. Bitioukov, V. Kachanov, D. Konstantinov, P. Mandrik, V. Petrov, R. Ryutin, S. Slabospitskii, A. Sobol, S. Troshin, N. Tyurin, A. Uzunian, A. Volkov, A. Babaev, A. Iuzhakov, V. Okhotnikov, V. Borchsh, V. Ivanchenko, E. Tcherniaev, P. Adzic, P. Cirkovic, M. Dordevic, P. Milenovic, J. Milosevic, M. Stojanovic, M. Aguilar-Benitez, J. Alcaraz Maestre, A. Álvarez Fernández, I. Bachiller, M. Barrio Luna, CristinaF. Bedoya, J. A. Brochero Cifuentes, C. A. Carrillo Montoya, M. Cepeda, M. Cerrada, N. Colino, B. DeLa Cruz, A. Delgado Peris, J. P. Fernández Ramos, J. Flix, M. C. Fouz, O. Gonzalez Lopez, S. Goy Lopez, J. M. Hernandez, M. I. Josa, D. Moran, Á. Navarro Tobar, A. Pérez-Calero Yzquierdo, J. Puerta Pelayo, I. Redondo, L. Romero, S. Sánchez Navas, M. S. Soares, A. Triossi, C. Willmott, C. Albajar, J. F. de Trocóniz, R. Reyes-Almanza, B. Alvarez Gonzalez, J. Cuevas, C. Erice, J. Fernandez Menendez, S. Folgueras, I. Gonzalez Caballero, J. R. González Fernández, E. Palencia Cortezon, V. Rodríguez Bouza, S. Sanchez Cruz, I. J. Cabrillo, A. Calderon, B. Chazin Quero, J. Duarte Campderros, M. Fernandez, P. J. Fernández Manteca, A. García Alonso, G. Gomez, C. Martinez Rivero, P. Martinez Ruiz del Arbol, F. Matorras, J. Piedra Gomez, C. Prieels, T. Rodrigo, A. Ruiz-Jimeno, L. Russo, L. Scodellaro, I. Vila, J. M. Vizan Garcia, K. Malagalage, W. G. D. Dharmaratna, N. Wickramage, D. Abbaneo, B. Akgun, E. Auffray, G. Auzinger, J. Baechler, P. Baillon, A. H. Ball, D. Barney, J. Bendavid, M. Bianco, A. Bocci, P. Bortignon, E. Bossini, C. Botta, E. Brondolin, T. Camporesi, A. Caratelli, G. Cerminara, E. Chapon, G. Cucciati, D. d’Enterria, A. Dabrowski, N. Daci, V. Daponte, A. David, O. Davignon, A. De Roeck, M. Deile, M. Dobson, M. Dünser, N. Dupont, A. Elliott-Peisert, N. Emriskova, F. Fallavollita, D. Fasanella, S. Fiorendi, G. Franzoni, J. Fulcher, W. Funk, S. Giani, D. Gigi, A. Gilbert, K. Gill, F. Glege, L. Gouskos, M. Gruchala, M. Guilbaud, D. Gulhan, J. Hegeman, C. Heidegger, Y. Iiyama, V. Innocente, T. James, P. Janot, O. Karacheban, J. Kaspar, J. Kieseler, M. Krammer, N. Kratochwil, C. Lange, P. Lecoq, C. Lourenço, L. Malgeri, M. Mannelli, A. Massironi, F. Meijers, S. Mersi, E. Meschi, F. Moortgat, M. Mulders, J. Ngadiuba, J. Niedziela, S. Nourbakhsh, S. Orfanelli, L. Orsini, F. Pantaleo, L. Pape, E. Perez, M. Peruzzi, A. Petrilli, G. Petrucciani, A. Pfeiffer, M. Pierini, F. M. Pitters, D. Rabady, A. Racz, M. Rieger, M. Rovere, H. Sakulin, J. Salfeld-Nebgen, C. Schäfer, C. Schwick, M. Selvaggi, A. Sharma, P. Silva, W. Snoeys, P. Sphicas, J. Steggemann, S. Summers, V. R. Tavolaro, D. Treille, A. Tsirou, G. P. Van Onsem, A. Vartak, M. Verzetti, W. D. Zeuner, L. Caminada, K. Deiters, W. Erdmann, R. Horisberger, Q. Ingram, H. C. Kaestli, D. Kotlinski, U. Langenegger, T. Rohe, S. A. Wiederkehr, M. Backhaus, P. Berger, N. Chernyavskaya, G. Dissertori, M. Dittmar, M. Donegà, C. Dorfer, T. A. Gómez Espinosa, C. Grab, D. Hits, W. Lustermann, R. A. Manzoni, M. T. Meinhard, F. Micheli, P. Musella, F. Nessi-Tedaldi, F. Pauss, G. Perrin, L. Perrozzi, S. Pigazzini, M. G. Ratti, M. Reichmann, C. Reissel, T. Reitenspiess, B. Ristic, D. Ruini, D. A. Sanz Becerra, M. Schönenberger, L. Shchutska, M. L. Vesterbacka Olsson, R. Wallny, D. H. Zhu, T. K. Aarrestad, C. Amsler, D. Brzhechko, M. F. Canelli, A. De Cosa, R. Del Burgo, B. Kilminster, S. Leontsinis, V. M. Mikuni, I. Neutelings, G. Rauco, P. Robmann, K. Schweiger, C. Seitz, Y. Takahashi, S. Wertz, A. Zucchetta, T. H. Doan, C. M. Kuo, W. Lin, A. Roy, S. S. Yu, P. Chang, Y. Chao, K. F. Chen, P. H. Chen, W.-S. Hou, Y. y. Li, R.-S. Lu, E. Paganis, A. Psallidas, A. Steen, B. Asavapibhop, C. Asawatangtrakuldee, N. Srimanobhas, N. Suwonjandee, A. Bat, F. Boran, A. Celik, S. Cerci, S. Damarseckin, Z. S. Demiroglu, F. Dolek, C. Dozen, I. Dumanoglu, G. Gokbulut, EmineGurpinar Guler, Y. Guler, I. Hos, C. Isik, E. E. Kangal, O. Kara, A. Kayis Topaksu, U. Kiminsu, G. Onengut, K. Ozdemir, S. Ozturk, A. E. Simsek, D. Sunar Cerci, U. G. Tok, S. Turkcapar, I. S. Zorbakir, C. Zorbilmez, B. Isildak, G. Karapinar, M. Yalvac, I. O. Atakisi, E. Gülmez, M. Kaya, O. Kaya, Ö. Özçelik, S. Tekten, E. A. Yetkin, A. Cakir, K. Cankocak, Y. Komurcu, S. Sen, B. Kaynak, S. Ozkorucuklu, B. Grynyov, L. Levchuk, E. Bhal, S. Bologna, J. J. Brooke, D. Burns, E. Clement, D. Cussans, H. Flacher, J. Goldstein, G. P. Heath, H. F. Heath, L. Kreczko, B. Krikler, S. Paramesvaran, B. Penning, T. Sakuma, S. Seif El Nasr-Storey, V. J. Smith, J. Taylor, A. Titterton, K. W. Bell, A. Belyaev, C. Brew, R. M. Brown, D. J. A. Cockerill, J. A. Coughlan, K. Harder, S. Harper, J. Linacre, K. Manolopoulos, D. M. Newbold, E. Olaiya, D. Petyt, T. Reis, T. Schuh, C. H. Shepherd-Themistocleous, A. Thea, I. R. Tomalin, T. Williams, W. J. Womersley, R. Bainbridge, P. Bloch, J. Borg, S. Breeze, O. Buchmuller, A. Bundock, GurpreetSingh CHAHAL, D. Colling, P. Dauncey, G. Davies, M. Della Negra, R. Di Maria, P. Everaerts, G. Hall, G. Iles, M. Komm, L. Lyons, A.-M. Magnan, S. Malik, A. Martelli, V. Milosevic, A. Morton, J. Nash, V. Palladino, M. Pesaresi, D. M. Raymond, A. Richards, A. Rose, E. Scott, C. Seez, A. Shtipliyski, M. Stoye, T. Strebler, A. Tapper, K. Uchida, T. Virdee, N. Wardle, D. Winterbottom, A. G. Zecchinelli, S. C. Zenz, J. E. Cole, P. R. Hobson, A. Khan, P. Kyberd, C. K. Mackay, I. D. Reid, L. Teodorescu, S. Zahid, K. Call, B. Caraway, J. Dittmann, K. Hatakeyama, C. Madrid, B. McMaster, N. Pastika, C. Smith, R. Bartek, A. Dominguez, R. Uniyal, A. M. Vargas Hernandez, A. Buccilli, S. I. Cooper, C. Henderson, P. Rumerio, C. West, A. Albert, D. Arcaro, Z. Demiragli, D. Gastler, C. Richardson, J. Rohlf, D. Sperka, I. Suarez, L. Sulak, D. Zou, G. Benelli, B. Burkle, X. Coubez, D. Cutts, Y. t. Duh, M. Hadley, U. Heintz, J. M. Hogan, K. H. M. Kwok, E. Laird, G. Landsberg, K. T. Lau, J. Lee, M. Narain, S. Sagir, R. Syarif, E. Usai, W. Y. Wong, D. Yu, W. Zhang, R. Band, C. Brainerd, R. Breedon, M. Calderon De La BarcaSanchez, M. Chertok, J. Conway, R. Conway, P. T. Cox, R. Erbacher, C. Flores, G. Funk, F. Jensen, W. Ko, O. Kukral, R. Lander, M. Mulhearn, D. Pellett, J. Pilot, M. Shi, D. Taylor, K. Tos, M. Tripathi, Z. Wang, F. Zhang, M. Bachtis, C. Bravo, R. Cousins, A. Dasgupta, A. Florent, J. Hauser, M. Ignatenko, N. Mccoll, W. A. Nash, S. Regnard, D. Saltzberg, C. Schnaible, B. Stone, V. Valuev, K. Burt, Y. Chen, R. Clare, J. W. Gary, S. M. A. Ghiasi Shirazi, G. Hanson, G. Karapostoli, O. R. Long, M. Olmedo Negrete, M. I. Paneva, W. Si, L. Wang, S. Wimpenny, B. R. Yates, Y. Zhang, J. G. Branson, P. Chang, S. Cittolin, S. Cooperstein, N. Deelen, M. Derdzinski, R. Gerosa, D. Gilbert, B. Hashemi, D. Klein, V. Krutelyov, J. Letts, M. Masciovecchio, S. May, S. Padhi, M. Pieri, V. Sharma, M. Tadel, F. Würthwein, A. Yagil, G. Zevi Della Porta, N. Amin, R. Bhandari, C. Campagnari, M. Citron, V. Dutta, M. Franco Sevilla, J. Incandela, B. Marsh, H. Mei, A. Ovcharova, H. Qu, J. Richman, U. Sarica, D. Stuart, S. Wang, D. Anderson, A. Bornheim, O. Cerri, I. Dutta, J. M. Lawhorn, N. Lu, J. Mao, H. B. Newman, T. Q. Nguyen, J. Pata, M. Spiropulu, J. R. Vlimant, S. Xie, Z. Zhang, R. Y. Zhu, M. B. Andrews, T. Ferguson, T. Mudholkar, M. Paulini, M. Sun, I. Vorobiev, M. Weinberg, J. P. Cumalat, W. T. Ford, E. MacDonald, T. Mulholland, R. Patel, A. Perloff, K. Stenson, K. A. Ulmer, S. R. Wagner, J. Alexander, Y. Cheng, J. Chu, A. Datta, A. Frankenthal, K. Mcdermott, J. R. Patterson, D. Quach, A. Ryd, S. M. Tan, Z. Tao, J. Thom, P. Wittich, M. Zientek, S. Abdullin, M. Albrow, M. Alyari, G. Apollinari, A. Apresyan, A. Apyan, S. Banerjee, L. A. T. Bauerdick, A. Beretvas, D. Berry, J. Berryhill, P. C. Bhat, K. Burkett, J. N. Butler, A. Canepa, G. B. Cerati, H. W. K. Cheung, F. Chlebana, M. Cremonesi, J. Duarte, V. D. Elvira, J. Freeman, Z. Gecse, E. Gottschalk, L. Gray, D. Green, S. Grünendahl, O. Gutsche, AllisonReinsvold Hall, J. Hanlon, R. M. Harris, S. Hasegawa, R. Heller, J. Hirschauer, B. Jayatilaka, S. Jindariani, M. Johnson, U. Joshi, T. Klijnsma, B. Klima, M. J. Kortelainen, B. Kreis, S. Lammel, J. Lewis, D. Lincoln, R. Lipton, M. Liu, T. Liu, J. Lykken, K. Maeshima, J. M. Marraffino, D. Mason, P. McBride, P. Merkel, S. Mrenna, S. Nahn, V. O’Dell, V. Papadimitriou, K. Pedro, C. Pena, G. Rakness, F. Ravera, L. Ristori, B. Schneider, E. Sexton-Kennedy, N. Smith, A. Soha, W. J. Spalding, L. Spiegel, S. Stoynev, J. Strait, N. Strobbe, L. Taylor, S. Tkaczyk, N. V. Tran, L. Uplegger, E. W. Vaandering, C. Vernieri, R. Vidal, M. Wang, H. A. Weber, D. Acosta, P. Avery, D. Bourilkov, A. Brinkerhoff, L. Cadamuro, V. Cherepanov, F. Errico, R. D. Field, S. V. Gleyzer, D. Guerrero, B. M. Joshi, M. Kim, J. Konigsberg, A. Korytov, K. H. Lo, K. Matchev, N. Menendez, G. Mitselmakher, D. Rosenzweig, K. Shi, J. Wang, S. Wang, X. Zuo, Y. R. Joshi, T. Adams, A. Askew, S. Hagopian, V. Hagopian, K. F. Johnson, R. Khurana, T. Kolberg, G. Martinez, T. Perry, H. Prosper, C. Schiber, R. Yohay, J. Zhang, M. M. Baarmand, M. Hohlmann, D. Noonan, M. Rahmani, M. Saunders, F. Yumiceva, M. R. Adams, L. Apanasevich, R. R. Betts, R. Cavanaugh, X. Chen, S. Dittmer, O. Evdokimov, C. E. Gerber, D. A. Hangal, D. J. Hofman, C. Mills, T. Roy, M. B. Tonjes, N. Varelas, J. Viinikainen, H. Wang, X. Wang, Z. Wu, M. Alhusseini, B. Bilki, K. Dilsiz, S. Durgut, R. P. Gandrajula, M. Haytmyradov, V. Khristenko, O. K. Köseyan, J.-P. Merlo, A. Mestvirishvili, A. Moeller, J. Nachtman, H. Ogul, Y. Onel, F. Ozok, A. Penzo, C. Snyder, E. Tiras, J. Wetzel, B. Blumenfeld, A. Cocoros, N. Eminizer, A. V. Gritsan, W. T. Hung, S. Kyriacou, P. Maksimovic, J. Roskes, M. Swartz, C. Baldenegro Barrera, P. Baringer, A. Bean, S. Boren, J. Bowen, A. Bylinkin, T. Isidori, S. Khalil, J. King, G. Krintiras, A. Kropivnitskaya, C. Lindsey, D. Majumder, W. Mcbrayer, N. Minafra, M. Murray, C. Rogan, C. Royon, S. Sanders, E. Schmitz, J. D. Tapia Takaki, Q. Wang, J. Williams, G. Wilson, S. Duric, A. Ivanov, K. Kaadze, D. Kim, Y. Maravin, D. R. Mendis, T. Mitchell, A. Modak, A. Mohammadi, F. Rebassoo, D. Wright, A. Baden, O. Baron, A. Belloni, S. C. Eno, Y. Feng, N. J. Hadley, S. Jabeen, G. Y. Jeng, R. G. Kellogg, A. C. Mignerey, S. Nabili, F. Ricci-Tam, M. Seidel, Y. H. Shin, A. Skuja, S. C. Tonwar, K. Wong, D. Abercrombie, B. Allen, A. Baty, R. Bi, S. Brandt, W. Busza, I. A. Cali, M. D’Alfonso, G. Gomez Ceballos, M. Goncharov, P. Harris, D. Hsu, M. Hu, M. Klute, D. Kovalskyi, Y.-J. Lee, P. D. Luckey, B. Maier, A. C. Marini, C. Mcginn, C. Mironov, S. Narayanan, X. Niu, C. Paus, D. Rankin, C. Roland, G. Roland, Z. Shi, G. S. F. Stephans, K. Sumorok, K. Tatar, D. Velicanu, J. Wang, T. W. Wang, B. Wyslouch, R. M. Chatterjee, A. Evans, S. Guts, P. Hansen, J. Hiltbrand, Sh. Jain, Y. Kubota, Z. Lesko, J. Mans, M. Revering, R. Rusack, R. Saradhy, N. Schroeder, M. A. Wadud, J. G. Acosta, S. Oliveros, K. Bloom, S. Chauhan, D. R. Claes, C. Fangmeier, L. Finco, F. Golf, R. Kamalieddin, I. Kravchenko, J. E. Siado, G. R. Snow, B. Stieger, W. Tabb, G. Agarwal, C. Harrington, I. Iashvili, A. Kharchilava, C. McLean, D. Nguyen, A. Parker, J. Pekkanen, S. Rappoccio, B. Roozbahani, G. Alverson, E. Barberis, C. Freer, Y. Haddad, A. Hortiangtham, G. Madigan, B. Marzocchi, D. M. Morse, T. Orimoto, L. Skinnari, A. Tishelman-Charny, T. Wamorkar, B. Wang, A. Wisecarver, D. Wood, S. Bhattacharya, J. Bueghly, T. Gunter, K. A. Hahn, N. Odell, M. H. Schmitt, K. Sung, M. Trovato, M. Velasco, R. Bucci, N. Dev, R. Goldouzian, M. Hildreth, K. Hurtado Anampa, C. Jessop, D. J. Karmgard, K. Lannon, W. Li, N. Loukas, N. Marinelli, I. Mcalister, F. Meng, Y. Musienko, R. Ruchti, P. Siddireddy, G. Smith, S. Taroni, M. Wayne, A. Wightman, M. Wolf, A. Woodard, J. Alimena, B. Bylsma, L. S. Durkin, B. Francis, C. Hill, W. Ji, A. Lefeld, T. Y. Ling, B. L. Winer, G. Dezoort, P. Elmer, J. Hardenbrook, N. Haubrich, S. Higginbotham, A. Kalogeropoulos, S. Kwan, D. Lange, M. T. Lucchini, J. Luo, D. Marlow, K. Mei, I. Ojalvo, J. Olsen, C. Palmer, P. Piroué, D. Stickland, C. Tully, S. Malik, S. Norberg, A. Barker, V. E. Barnes, S. Das, L. Gutay, M. Jones, A. W. Jung, A. Khatiwada, B. Mahakud, D. H. Miller, G. Negro, N. Neumeister, C. C. Peng, S. Piperov, H. Qiu, J. F. Schulte, N. Trevisani, F. Wang, R. Xiao, W. Xie, T. Cheng, J. Dolen, N. Parashar, U. Behrens, K. M. Ecklund, S. Freed, F. J. M. Geurts, M. Kilpatrick, Arun Kumar, W. Li, B. P. Padley, R. Redjimi, J. Roberts, J. Rorie, W. Shi, A. G. Stahl Leiton, Z. Tu, A. Zhang, A. Bodek, P. de Barbaro, R. Demina, J. L. Dulemba, C. Fallon, T. Ferbel, M. Galanti, A. Garcia-Bellido, O. Hindrichs, A. Khukhunaishvili, E. Ranken, R. Taus, B. Chiarito, J. P. Chou, A. Gandrakota, Y. Gershtein, E. Halkiadakis, A. Hart, M. Heindl, E. Hughes, S. Kaplan, I. Laflotte, A. Lath, R. Montalvo, K. Nash, M. Osherson, H. Saka, S. Salur, S. Schnetzer, S. Somalwar, R. Stone, S. Thomas, H. Acharya, A. G. Delannoy, S. Spanier, O. Bouhali, M. Dalchenko, M. De Mattia, A. Delgado, S. Dildick, R. Eusebi, J. Gilmore, T. Huang, T. Kamon, H. Kim, S. Luo, S. Malhotra, D. Marley, R. Mueller, D. Overton, L. Perniè, D. Rathjens, A. Safonov, N. Akchurin, J. Damgov, F. De Guio, V. Hegde, S. Kunori, K. Lamichhane, S. W. Lee, T. Mengke, S. Muthumuni, T. Peltola, S. Undleeb, I. Volobouev, Z. Wang, A. Whitbeck, S. Greene, A. Gurrola, R. Janjam, W. Johns, C. Maguire, A. Melo, H. Ni, K. Padeken, F. Romeo, P. Sheldon, S. Tuo, J. Velkovska, M. Verweij, M. W. Arenton, P. Barria, B. Cox, G. Cummings, J. Hakala, R. Hirosky, M. Joyce, A. Ledovskoy, C. Neu, B. Tannenwald, Y. Wang, E. Wolfe, F. Xia, R. Harr, P. E. Karchin, N. Poudyal, J. Sturdy, P. Thapa, T. Bose, J. Buchanan, C. Caillol, D. Carlsmith, S. Dasu, I. De Bruyn, L. Dodd, C. Galloni, H. He, M. Herndon, A. Hervé, U. Hussain, A. Lanaro, A. Loeliger, K. Long, R. Loveless, J. Madhusudanan Sreekala, D. Pinna, T. Ruggles, A. Savin, V. Sharma, W. H. Smith, D. Teague, S. Trembath-reichert, CMS Collaboration

**Affiliations:** 1 Yerevan Physics Institute, Yerevan, Armenia; 2 Institut für Hochenergiephysik, Wien, Austria; 3 Institute for Nuclear Problems, Minsk, Belarus; 4 Universiteit Antwerpen, Antwerpen, Belgium; 5 Vrije Universiteit Brussel, Brussel, Belgium; 6 Université Libre de Bruxelles, Bruxelles, Belgium; 7 Ghent University, Ghent, Belgium; 8 Université Catholique de Louvain, Louvain-la-Neuve, Belgium; 9 Centro Brasileiro de Pesquisas Fisicas, Rio de Janeiro, Brazil; 10 Universidade do Estado do Rio de Janeiro, Rio de Janeiro, Brazil; 11 Universidade Estadual Paulista , Universidade Federal do ABC, São Paulo, Brazil; 12 Institute for Nuclear Research and Nuclear Energy, Bulgarian Academy of Sciences, Sofia, Bulgaria; 13 University of Sofia, Sofia, Bulgaria; 14 Beihang University, Beijing, China; 15 Department of Physics, Tsinghua University, Beijing, China; 16 Institute of High Energy Physics, Beijing, China; 17 State Key Laboratory of Nuclear Physics and Technology, Peking University, Beijing, China; 18 Zhejiang University, Hangzhou, China; 19 Universidad de Los Andes, Bogota, Colombia; 20 Universidad de Antioquia, Medellin, Colombia; 21 University of Split, Faculty of Electrical Engineering, Mechanical Engineering and Naval Architecture, Split, Croatia; 22 University of Split, Faculty of Science, Split, Croatia; 23 Institute Rudjer Boskovic, Zagreb, Croatia; 24 University of Cyprus, Nicosia, Cyprus; 25 Charles University, Prague, Czech Republic; 26 Escuela Politecnica Nacional, Quito, Ecuador; 27 Universidad San Francisco de Quito, Quito, Ecuador; 28 Academy of Scientific Research and Technology of the Arab Republic of Egypt, Egyptian Network of High Energy Physics, Cairo, Egypt; 29 National Institute of Chemical Physics and Biophysics, Tallinn, Estonia; 30 Department of Physics, University of Helsinki, Helsinki, Finland; 31 Helsinki Institute of Physics, Helsinki, Finland; 32 Lappeenranta University of Technology, Lappeenranta, Finland; 33 IRFU, CEA, Université Paris-Saclay, Gif-sur-Yvette, France; 34 Laboratoire Leprince-Ringuet, CNRS/IN2P3, Ecole Polytechnique, Institut Polytechnique de Paris, Palaiseau, France; 35 Université de Strasbourg, CNRS, IPHC UMR 7178, Strasbourg, France; 36 Centre de Calcul de l’Institut National de Physique Nucleaire et de Physique des Particules, CNRS/IN2P3, Villeurbanne, France; 37 Université de Lyon, Université Claude Bernard Lyon 1, CNRS-IN2P3, Institut de Physique Nucléaire de Lyon, Villeurbanne, France; 38 Georgian Technical University, Tbilisi, Georgia; 39 Tbilisi State University, Tbilisi, Georgia; 40 RWTH Aachen University, I. Physikalisches Institut, Aachen, Germany; 41 RWTH Aachen University, III. Physikalisches Institut A, Aachen, Germany; 42 RWTH Aachen University, III. Physikalisches Institut B, Aachen, Germany; 43 Deutsches Elektronen-Synchrotron, Hamburg, Germany; 44 University of Hamburg, Hamburg, Germany; 45 Karlsruher Institut fuer Technologie, Karlsruhe, Germany; 46 Institute of Nuclear and Particle Physics (INPP), NCSR Demokritos, Aghia Paraskevi, Greece; 47 National and Kapodistrian University of Athens, Athens, Greece; 48 National Technical University of Athens, Athens, Greece; 49 University of Ioánnina, Ioánnina, Greece; 50 MTA-ELTE Lendület CMS Particle and Nuclear Physics Group, Eötvös Loránd University, Budapest, Hungary; 51 Wigner Research Centre for Physics, Budapest, Hungary; 52 Institute of Nuclear Research ATOMKI, Debrecen, Hungary; 53 Institute of Physics, University of Debrecen, Debrecen, Hungary; 54 Eszterhazy Karoly University, Karoly Robert Campus, Gyongyos, Hungary; 55 Indian Institute of Science (IISc), Bangalore, India; 56 National Institute of Science Education and Research, HBNI, Bhubaneswar, India; 57 Panjab University, Chandigarh, India; 58 University of Delhi, Delhi, India; 59 Saha Institute of Nuclear Physics, HBNI, Kolkata, India; 60 Indian Institute of Technology Madras, Madras, India; 61 Bhabha Atomic Research Centre, Mumbai, India; 62 Tata Institute of Fundamental Research-A, Mumbai, India; 63 Tata Institute of Fundamental Research-B, Mumbai, India; 64 Indian Institute of Science Education and Research (IISER), Pune, India; 65 Institute for Research in Fundamental Sciences (IPM), Tehran, Iran; 66 University College Dublin, Dublin, Ireland; 67 INFN Sezione di Bari , Università di Bari , Politecnico di Bari, Bari, Italy; 68 INFN Sezione di Bologna , Università di Bologna, Bologna, Italy; 69 INFN Sezione di Catania , Università di Catania, Catania, Italy; 70 INFN Sezione di Firenze , Università di Firenze, Firenze, Italy; 71 INFN Laboratori Nazionali di Frascati, Frascati, Italy; 72 INFN Sezione di Genova , Università di Genova, Genova, Italy; 73 INFN Sezione di Milano-Bicocca , Università di Milano-Bicocca, Milano, Italy; 74 INFN Sezione di Napoli , Università di Napoli ’Federico II’ , Napoli; Italy, Università della Basilicata , Potenza, Italy, Università G. Marconi, Roma, Italy; 75 INFN Sezione di Padova , Università di Padova , Padova; Italy, Università di Trento, Trento, Italy; 76 INFN Sezione di Pavia , Università di Pavia, Pavia, Italy; 77 INFN Sezione di Perugia , Università di Perugia, Perugia, Italy; 78 INFN Sezione di Pisa , Università di Pisa , Scuola Normale Superiore di Pisa, Pisa, Italy; 79 INFN Sezione di Roma , Sapienza Università di Roma, Rome, Italy; 80 INFN Sezione di Torino , Università di Torino , Torino; Italy, Università del Piemonte Orientale, Novara, Italy; 81 INFN Sezione di Trieste , Università di Trieste, Trieste, Italy; 82 Kyungpook National University, Daegu, Korea; 83 Chonnam National University, Institute for Universe and Elementary Particles, Kwangju, Korea; 84 Hanyang University, Seoul, Korea; 85 Korea University, Seoul, Korea; 86 Department of Physics, Kyung Hee University, Seoul, Korea; 87 Sejong University, Seoul, Korea; 88 Seoul National University, Seoul, Korea; 89 University of Seoul, Seoul, Korea; 90 Sungkyunkwan University, Suwon, Korea; 91 Riga Technical University, Riga, Latvia; 92 Vilnius University, Vilnius, Lithuania; 93 National Centre for Particle Physics, Universiti Malaya, Kuala Lumpur, Malaysia; 94 Universidad de Sonora (UNISON), Hermosillo, Mexico; 95 Centro de Investigacion y de Estudios Avanzados del IPN, Mexico City, Mexico; 96 Universidad Iberoamericana, Mexico City , Mexico; 97 Benemerita Universidad Autonoma de Puebla, Puebla, Mexico; 98 Universidad Autónoma de San Luis Potosí, San Luis Potosí, Mexico; 99 University of Montenegro, Podgorica, Montenegro; 100 University of Auckland, Auckland, New Zealand; 101 University of Canterbury, Christchurch, New Zealand; 102 National Centre for Physics, Quaid-I-Azam University, Islamabad, Pakistan; 103 AGH University of Science and Technology Faculty of Computer Science, Electronics and Telecommunications, Krakow, Poland; 104 National Centre for Nuclear Research, Swierk, Poland; 105 Institute of Experimental Physics, Faculty of Physics, University of Warsaw, Warsaw, Poland; 106 Laboratório de Instrumentação e Física Experimental de Partículas, Lisbon, Portugal; 107 Joint Institute for Nuclear Research, Dubna, Russia; 108 Petersburg Nuclear Physics Institute, Gatchina (St. Petersburg), Russia; 109 Institute for Nuclear Research, Moscow, Russia; 110 Institute for Theoretical and Experimental Physics named by A.I. Alikhanov of NRC ‘Kurchatov Institute’, Moscow, Russia; 111 Moscow Institute of Physics and Technology, Moscow, Russia; 112 National Research Nuclear University ’Moscow Engineering Physics Institute’ (MEPhI), Moscow, Russia; 113 P.N. Lebedev Physical Institute, Moscow, Russia; 114 Skobeltsyn Institute of Nuclear Physics, Lomonosov Moscow State University, Moscow, Russia; 115 Novosibirsk State University (NSU), Novosibirsk, Russia; 116 Institute for High Energy Physics of National Research Centre ‘Kurchatov Institute’, Protvino, Russia; 117 National Research Tomsk Polytechnic University, Tomsk, Russia; 118 Tomsk State University, Tomsk, Russia; 119 University of Belgrade: Faculty of Physics and VINCA Institute of Nuclear Sciences, Belgrade, Serbia; 120 Centro de Investigaciones Energéticas Medioambientales y Tecnológicas (CIEMAT), Madrid, Spain; 121 Universidad Autónoma de Madrid, Madrid, Spain; 122 Universidad de Oviedo, Instituto Universitario de Ciencias y Tecnologías Espaciales de Asturias (ICTEA), Oviedo, Spain; 123 Instituto de Física de Cantabria (IFCA), CSIC-Universidad de Cantabria, Santander, Spain; 124 University of Colombo, Colombo, Sri Lanka; 125 Department of Physics, University of Ruhuna, Matara, Sri Lanka; 126 CERN, European Organization for Nuclear Research, Geneva, Switzerland; 127 Paul Scherrer Institut, Villigen, Switzerland; 128 ETH Zurich - Institute for Particle Physics and Astrophysics (IPA), Zurich, Switzerland; 129 Universität Zürich, Zurich, Switzerland; 130 National Central University, Chung-Li, Taiwan; 131 National Taiwan University (NTU), Taipei, Taiwan; 132 Department of Physics, Chulalongkorn University, Faculty of Science, Bangkok, Thailand; 133 Physics Department Science and Art Faculty, Çukurova University, Adana, Turkey; 134 Physics Department, Middle East Technical University, Ankara, Turkey; 135 Bogazici University, Istanbul, Turkey; 136 Istanbul Technical University, Istanbul, Turkey; 137 Istanbul University, Istanbul, Turkey; 138 Institute for Scintillation Materials of National Academy of Science of Ukraine, Kharkov, Ukraine; 139 National Scientific Center, Kharkov Institute of Physics and Technology, Kharkov, Ukraine; 140 University of Bristol, Bristol, United Kingdom; 141 Rutherford Appleton Laboratory, Didcot, United Kingdom; 142 Imperial College, London, United Kingdom; 143 Brunel University, Uxbridge, United Kingdom; 144 Baylor University, Waco, USA; 145 Catholic University of America, Washington, DC USA; 146 The University of Alabama, Tuscaloosa, USA; 147 Boston University, Boston, USA; 148 Brown University, Providence, USA; 149 University of California, Davis, Davis, USA; 150 University of California, Los Angeles, USA; 151 University of California, Riverside, Riverside, USA; 152 University of California, San Diego, La Jolla USA; 153 Department of Physics, University of California, Santa Barbara, Santa Barbara, USA; 154 California Institute of Technology, Pasadena, USA; 155 Carnegie Mellon University, Pittsburgh, USA; 156 University of Colorado Boulder, Boulder, USA; 157 Cornell University, Ithaca, USA; 158 Fermi National Accelerator Laboratory, Batavia, USA; 159 University of Florida, Gainesville, USA; 160 Florida International University, Miami, USA; 161 Florida State University, Tallahassee, USA; 162 Florida Institute of Technology, Melbourne, USA; 163 University of Illinois at Chicago (UIC), Chicago, USA; 164 The University of Iowa, Iowa City, USA; 165 Johns Hopkins University, Baltimore, USA; 166 The University of Kansas, Lawrence, USA; 167 Kansas State University, Manhattan, USA; 168 Lawrence Livermore National Laboratory, Livermore, USA; 169 University of Maryland, College Park, USA; 170 Massachusetts Institute of Technology, Cambridge, USA; 171 University of Minnesota, Minneapolis, USA; 172 University of Mississippi, Oxford, USA; 173 University of Nebraska-Lincoln, Lincoln, USA; 174 State University of New York at Buffalo, Buffalo, USA; 175 Northeastern University, Boston, USA; 176 Northwestern University, Evanston, USA; 177 University of Notre Dame, Notre Dame, USA; 178 The Ohio State University, Columbus, USA; 179 Princeton University, Princeton, USA; 180 University of Puerto Rico, Mayaguez, USA; 181 Purdue University, West Lafayette, USA; 182 Purdue University Northwest, Hammond, USA; 183 Rice University, Houston, USA; 184 University of Rochester, Rochester, USA; 185 Rutgers, The State University of New Jersey, Piscataway, USA; 186 University of Tennessee, Knoxville, USA; 187 Texas A&M University, College Station, USA; 188 Texas Tech University, Lubbock, USA; 189 Vanderbilt University, Nashville, USA; 190 University of Virginia, Charlottesville, USA; 191 Wayne State University, Detroit, USA; 192 University of Wisconsin - Madison, Madison, WI USA; 193 CERN, 1211 Geneva 23, Switzerland

**Keywords:** CMS, b jets, Higgs boson, Jet energy, Jet resolution, Deep learning

## Abstract

We describe a method to obtain point and dispersion estimates for the energies of jets arising from b quarks produced in proton–proton collisions at an energy of $\sqrt{s}=13\,\text {TeV}$ at the CERN LHC. The algorithm is trained on a large sample of simulated b jets and validated on data recorded by the CMS detector in 2017 corresponding to an integrated luminosity of 41 $\,\text {fb}^{-1}$. A multivariate regression algorithm based on a deep feed-forward neural network employs jet composition and shape information, and the properties of reconstructed secondary vertices associated with the jet. The results of the algorithm are used to improve the sensitivity of analyses that make use of b jets in the final state, such as the observation of Higgs boson decay to $\hbox {b}\bar{\hbox {b}}$.

## Introduction

Following the discovery of the 125 $\text {GeV}$ Higgs boson reported by the ATLAS and CMS Collaborations at the CERN LHC in 2012 [1–3], a rich research program was established to probe this new particle. The program includes the measurement of all production and decay modes that are accessible at the LHC. The decay of the Higgs boson into a pair of vector bosons was established with a statistical significance higher than five standard deviations individually for photon, Z and W pairs using data collected at the LHC from 2011 to 2013 at center-of-mass energies of $\sqrt{s}=7$ and 8 $\,\text {TeV}$ [4–9]. A few years later, the combination of CMS data sets collected at 8 and 13 $\text {TeV}$ was used to report the observation of Higgs boson decay to a pair of $\tau$ leptons [10], followed by the observation of the associated production of a Higgs boson with a top quark–antiquark pair ($\hbox {t}\bar{\hbox {t}}$) [11, 12].

Higgs boson decay to a b quark–antiquark pair ($\hbox {b}\bar{\hbox {b}}$) was only recently announced by the CMS [13] and ATLAS [14] collaborations, despite it being the dominant decay mode. This is because of the challenges associated with separating the signal from the large background of $\hbox {b}\bar{\hbox {b}}$ produced by quantum chromodynamics (QCD) processes. Good resolution of the reconstructed invariant mass of Higgs boson candidates is necessary to have a more favorable signal-to-background ratio. This is achieved in CMS by the method described in this paper, based on a deep neural network (DNN) that estimates the energy of jets originating from b quarks (b jets). Similar algorithms, using neural networks, were previously used by the CDF Collaboration at the Tevatron [15, 16], and BDT-based energy regressions were used earlier by the CMS Collaboration to estimate the energy of b jets [17].

The approach described in this paper is to use a regression algorithm that is implemented in a feed-forward neural network with six hidden layers trained on a very large data set, consisting of Monte Carlo (MC) simulated b jets. The algorithm has a considerably larger modeling capability than those used previously. This approach was made possible by leveraging recent advances in hardware accelerators, such as graphics processing units (GPU), and in modern packages for automatic differentiation to handle the otherwise expensive computations involved in this task. A minimization of a loss function that combines a Huber [18] and two quantile [19] loss terms enables simultaneous training of point and dispersion estimators of the regression target without making any assumptions about the functional form of its distribution. The point estimator is used as a correction of the measured b jet energy, while dispersion estimators are used to build a jet-by-jet resolution estimate. The CMS collaboration had previously developed a BDT-based approach to estimate the energy and per-object resolution [20–22]. This can be achieved by training separate regressions to obtain energy and per-object resolution estimators, or by means of a semiparametric regression [20, 21]. For a semiparametric regression, the training relies on the knowledge of the analytical shape of the target distribution. The novel characteristic of the algorithm described in this paper is the simultaneous training of the point and dispersion estimators without reference to an ansatz distribution for the regression target. This method is validated on data collected by the CMS detector in 2017.

In the following, Sect. 2 and Sect. 3 describe the CMS detector and the data sets used for this work. The regression problem and the inputs are described in Sect. 4. In Sect. 5, the loss function is introduced, while the DNN architecture and its training are summarized in Sect. 6. Finally, the results are presented in Sect. 7, followed by the summary in Sect. 8.

## The CMS Detector

The central feature of the CMS detector is a superconducting solenoid of 6 m internal diameter, providing a magnetic field of 3.8 T. Within the solenoid volume are a silicon pixel and strip tracker, a lead tungstate crystal electromagnetic calorimeter (ECAL), and a brass and scintillator hadron calorimeter (HCAL), each composed of a barrel and two endcap sections. Forward calorimeters extend the pseudorapidity ($\eta$) coverage provided by the barrel and endcap detectors. Muons are detected in gas-ionization chambers embedded in the steel flux-return yoke outside the solenoid. A detailed description of the apparatus, together with a definition of the coordinate system used and the relevant kinematic variables, can be found in Ref. [23].

The particle-flow (PF) algorithm [24] used by CMS aims to reconstruct and identify each individual particle in an event, with an optimized combination of information from the various elements of the CMS detector. Photon energies are obtained from ECAL data. The candidate vertex with the largest value of summed physics-object $p_{\mathrm {T}} ^2$ is taken to be the primary proton–proton ($\hbox {p}{}{} \hbox {p}{}{}$) interaction vertex. The energy of each electron in the event is determined from a combination of the electron momentum at the primary interaction vertex, as determined by the tracker, the energy of the corresponding ECAL cluster, and the energy sum of all bremsstrahlung photons spatially compatible with having originated from the electron. The momentum of each muon is obtained via the curvature of the corresponding track. The energy of each charged hadron is determined from a combination of momentum measured in the tracker and the matching ECAL and HCAL energy deposits, corrected for zero-suppression effects and for the response function of the calorimeters to hadronic showers. Finally, for a neutral hadron, the energy is obtained from the corresponding HCAL corrected energies. The anti-$k_{\mathrm {T}}$ algorithm [25, 26] with a distance parameter of 0.4 is applied offline to the full set of PF candidates to cluster them into jets. The jet momentum is determined by the vectorial sum of all particle momenta in the jet. The jet energy resolution typically amounts to 15–20% at 30 $\text {GeV}$, 10% at 100 $\text {GeV}$, and 5% at 1 $\text {TeV}$[27].

Additional $\hbox {p}{}{} \hbox {p}{}{}$ interactions within the same or nearby bunch crossings (pileup) can contribute unrelated particles to the jet. To mitigate the effects of pileup, charged particles with tracks originating from pileup vertices are discarded before jet reconstruction. Then, the residual contamination from neutral particles and charged particles without reconstructed tracks is estimated for each event and subtracted from the jet energy. Jet energy corrections are derived from simulation to bring the measured average response for jets in line with particle-level jets. Neutrinos are not included in the clustering of particle-level jets. In situ measurements of the transverse momentum balance in dijet, photon+jet, $\hbox {Z}{}{}$+jet, and multijet events are used to account for residual differences between the jet energy scales in data and simulation [28]. We refer to this correction algorithm as the baseline algorithm.

## Data Sets

The DNN was trained on 100 million b jets from a simulated sample of $\hbox {t}\bar{\hbox {t}}$ events produced in pp collisions at $\sqrt{s}=13\,\text {TeV}$, generated at next-to-leading-order (NLO) accuracy in perturbative QCD (pQCD) with the powheg v2 program [29]. Predictions of the model were then tested on simulated events with b jets coming from a variety of physical processes to validate performance in all relevant kinematic regions. To this end, b jets from the decay of Higgs bosons produced in association with a Z boson, $\hbox {Z}{}{} (\rightarrow \ell ^+\ell ^-)\hbox {H}{}{} (\rightarrow \hbox {b}\bar{\hbox {b}})$, where $\ell$ is an electron or a muon, were generated with the MadGraph 5_amc@nlo generator [30] at NLO pQCD accuracy. Additionally, b jets from the decay of Higgs boson pairs produced either from gluon fusion or in the decay of a new, spin-0 resonance, with one Higgs boson decaying to a b quark-antiquark pair and the other to a pair of photons, $\hbox {H}{}{} (\rightarrow \hbox {b}\bar{\hbox {b}})\hbox {H}{}{} (\rightarrow {\upgamma }{}{} {\upgamma }{}{})$, were generated with MadGraph 5_amc@nlo at leading-order accuracy in pQCD.

Two definitions of jets are used in this study: “generator-level jets”, clustered from stable particles produced by the MC generator that include the contribution from the neutrino’s momentum, and “reconstructed jets”, clustered from reconstructed particle-flow candidates. The reconstructed b jets were matched to generated b jets to avoid contamination by light flavored jets. For each reconstructed jet, the corresponding generator-level jet is found by spatial matching in the $\eta -\phi$ plane by requiring the distance $\varDelta R = \sqrt{\smash [b]{(\varDelta \eta )^2+(\varDelta \phi )^2}}$ (where $\phi$ is the azimuthal angle in radians) to be $\varDelta R < 0.4$. The reconstructed b jets were then selected by applying a minimum threshold for transverse momentum ($p_{\mathrm {T}} ^\text {reco}> 15$ $\,\text {GeV}$, $p_{\mathrm {T}} ^{\text {gen}}> 15$ $\,\text {GeV}$) and by requiring the pseudorapidity of the central axis of the reconstructed jet to be within the tracker acceptance ($|\eta | < 2.4$).

Finally, to validate the regression model on data, the output of the DNN for simulated b jets was compared to that obtained for b jets recorded by the CMS detector. The events used for this validation were recorded in 2017 with triggers [31] that require the presence of at least one lepton. This data set, corresponding to an integrated luminosity of 41 $\,\text {fb}^{-1}$, was further enriched in Z bosons produced in association with b jets. The corresponding simulated events come from a sample of Z bosons and up to two additional partons generated with MadGraph 5_amc@nlo at NLO accuracy in pQCD.

For all simulated events, pythia 8.2 [32] with the CP5 tune [33] is used for parton showering and hadronization. The CMS detector response is simulated by the Geant4 [34] package, and simulated pileup interactions are added to the hard-scattering process to match the distribution of pileup interactions observed in data, for which the observed mean number of interactions per bunch crossing is 32.

## Energy Regression and Input Features

In comparison to jets arising from light-flavor quarks or gluons, jets arising from b quarks have special characteristics that call for dedicated energy corrections. In particular, b jets contain b hadrons that can often decay to a final state with a charged lepton and a neutrino. The neutrinos, which only interact via the weak force, escape detection, leading to an underestimate of the b jet energy, with a corresponding degradation of energy resolution. As described in Sect. 2, the jet energy is reconstructed by clustering its constituents within a given distance parameter. Compared to jets originating from light-flavor quarks and gluons, b jets, because of their higher mass, tend to spread radially over a wider area in the $\eta$-$\phi$ plane. This often leads to leakage of energy outside of the jet clustering region, further impacting the jet energy response and resolution.

**Fig. 1:**
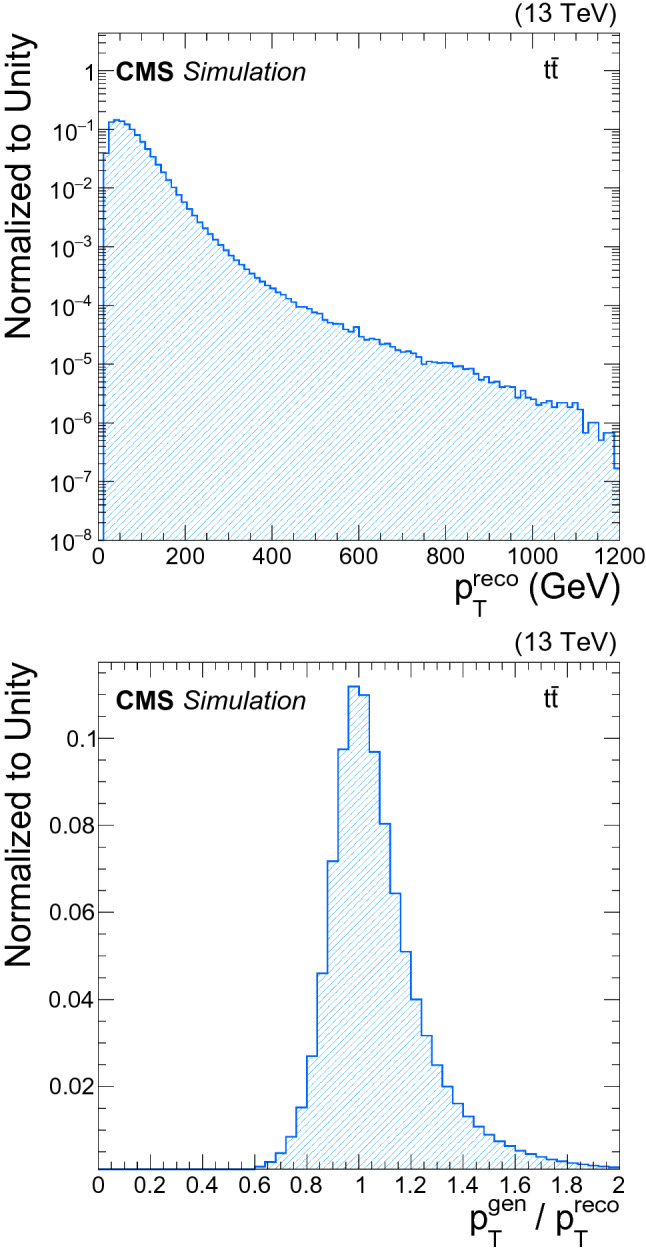
(upper) The $p_{\mathrm {T}} ^\text {reco}$ distribution for reconstructed b jets in an MC $\hbox {t}\bar{\hbox {t}}$sample. (lower) Distribution of the regression target for the MC $\hbox {t}\bar{\hbox {t}}$training sample

The b jets used for the DNN training come from a sample of simulated top quark events. The top quark decays before hadronising with a branching fraction close to unity into a b jet and a W boson. At LHC energies, it provides a source of b jets that spans a large transverse momentum ($p_{\mathrm {T}}$) spectrum and covers the full $\eta$ acceptance of the detector. The $p_{\mathrm {T}} ^\text {reco}$ value is corrected with the baseline algorithm as described in Sect. 2. Figure 1 (upper) shows the distribution of $p_{\mathrm {T}} ^\text {reco}$, for the selected b jets.

The regression target, *y*, used in this study is defined as the ratio of the transverse momentum of the generator-level jet, $p_{\mathrm {T}} ^{\text {gen}}$, to that of the reconstructed jet, $p_{\mathrm {T}} ^\text {reco}$, applying the baseline jet energy corrections. Using this definition rather than using $p_{\mathrm {T}} ^{\text {gen}}$ directly has the effect of greatly reducing the variance of the target while producing a numerical value of order 1. The distribution of the target for b jets from an MC simulated $\hbox {t}\bar{\hbox {t}}$ sample is shown in Fig. 1 (lower). To improve the convergence of the training of the DNN, the target is further standardized by subtracting its median value and dividing it by its standard deviation.

The DNN training inputs provide information about the kinematics, shape, and composition of reconstructed jets. The inputs consist of the following features:

- jet kinematics: jet $p_{\mathrm {T}}$, $\eta$, mass, and transverse mass, defined as $\sqrt{\smash [b]{ E^2 - p_z ^2} }$;
- information about pileup interactions: the median energy density in the event, $\rho$, corresponding to the amount of transverse momentum per unit area that is due to overlapping collisions [35];
- information about semileptonic decays of b hadrons when an electron or muon candidate is clustered within a jet: the transverse component of lepton momentum perpendicular to the jet axis, the distance $\varDelta R = \sqrt{\smash [b]{(\varDelta \eta )^2+(\varDelta \phi )^2}}$, and a categorical variable that encodes information about the lepton candidate’s flavor;
- information about the secondary vertex, selected as the highest $p_{\mathrm {T}}$ displaced vertex linked to the jet: number of tracks associated to the vertex, transverse momentum, and mass (computed assigning the pion mass to all reconstructed tracks forming the secondary vertex); the distance between the collision vertex and the secondary vertex computed in three-dimensional space with its associated uncertainty [36, 37];
- jet composition: largest $p_{\mathrm {T}}$ value of any charged hadron candidates, fractions of energy carried by jet constituents; namely charged hadrons, neutral hadrons, muons, and an electromagnetic component coming from electrons and photons. These fractions are computed for the whole jet, and separately in five rings of $\varDelta R$ around the jet axis ($\varDelta R =$ 0–0.05, 0.05–0.1, 0.1–0.2, 0.2–0.3, 0.3–0.4);
- multiplicity of PF candidates clustered to form the jet;
- information about jet energy sharing among the jet constituents computed as
  1 $$\begin{aligned} \frac{\sqrt{\sum _ip_{\text T,i}^2}}{{\sum _ip_{\text T,i}}}, \end{aligned}$$
  where *i* runs over all jet constituents.

This results in a total of 41 input features. No additional preprocessing is performed, apart from the input normalization provided by batch normalization [38] at the input layer of the DNN.

## Loss Function

A possible approach to such a regression problem is to develop separate dedicated regressions to obtain energy and per-object resolution estimators. If the target distribution can be parametrized analytically, one can use a semiparametric regression to obtain estimates of the function parameters. This method has been used by the CMS collaboration to estimate the energy and resolution of electron and photon candidates [20, 21]. Whereas for the photon and electron candidates, the energy response can be parametrized by an analytically integrable function, this is less straightforward for b jets, making such an approach to the problem more expensive computationally. An alternative approach is to simultaneously obtain point and dispersion estimates of the b jet energy by defining a loss function that is completely agnostic to the target distribution. The correction to be applied to the reconstructed b jet energy can be obtained as the estimated mean, while the per-jet b jet energy resolution can be estimated as half the difference of the 75 and 25% quantiles. Therefore, the regression loss function should provide the mean estimator (${\hat{y}}$), and the 25 and 75% quantiles of the target distribution.

The Huber loss function is employed to learn the mean of the target distribution via a minimization process. It is preferable to the mean squared error because of its reduced sensitivity to the tails of the target distribution. It is defined as:

2 $$\begin{aligned} H_{\delta }(z) = {\left\{ \begin{array}{ll} \frac{1}{2} z ^2, &{}\text {if }|z| < \delta ;\\ \delta |z| - \frac{1}{2}\delta ^2, &{}\text {otherwise,} \end{array}\right. } \end{aligned}$$

where $z = y -{\hat{y}}$, and $\delta$ is set to 1 in our case. To estimate the 25 and 75% quantiles of the target distribution, the quantile loss function is used:

3 $$\begin{aligned} \rho _{\tau }(z) = {\left\{ \begin{array}{ll} \tau z, &{}\text {if }z > 0;\\ (\tau - 1) z, &{}\text {otherwise,} \end{array}\right. } \end{aligned}$$

where $\tau$ = 0.25 (0.75) corresponds to the 25 (75)% quantile.

The complete loss function can then be written as:

4 $$\begin{aligned} \text {loss}({\hat{y}},{\hat{y}}_{25\%},{\hat{y}}_{75\%}) = E_{(x,y) \sim p(x,y)} [H_{1}( y - {\hat{y}}(x) )+\rho _{0.25}( y - {\hat{y}}_{25\%}(x)) +\rho _{0.75}( y -{\hat{y}}_{75\%}(x))], \end{aligned}$$

where $E_{(x,y) \sim p(x,y)}$ denotes the expectation value when sampling (*x*, *y*) on the distribution *p*(*x*, *y*), *x* denotes the set of input features, and *p*(*x*, *y*) is the joint distribution of the input features and the target variables *y* in the training sample. The symbols ${\hat{y}}(x)$, ${\hat{y}}_{25\%}(x)$, and ${\hat{y}}_{75\%}(x)$ denote the DNN outputs: ${\hat{y}}(x)$ is the mean estimator, and ${\hat{y}}_{25\%}(x)$ and ${\hat{y}}_{75\%}(x)$ are the 25 and 75% quantile estimators, respectively.

## Neural Network Architecture

The model used for this study is a feed-forward, fully connected DNN with 6 hidden layers, 41 input features, and 3 outputs: the energy correction and the 25 and 75% quantiles. As mentioned above, a batch normalization layer is applied at the DNN input.

Each hidden layer of the DNN is built from the following components:

- Dense layer: defined as a linear combination of all outputs from the previous layer.
- Batch normalization layer: to transform the inputs to zero-mean and unit-variance.
- Dropout unit: an operation that zeroes a fixed fraction of randomly chosen nodes during the training, used as a regularization handle. The dropout rate is one of the optimized hyperparameters of the DNN.
- Activation unit: we chose the “Leaky” Rectified Linear Unit (LReLU) [39]:
  5 $$\begin{aligned} \text {LReLU}(x) = {\left\{ \begin{array}{ll} x, &{}\hbox { if}\ x \ge 0;\\ \beta x, &{}\hbox { if}\ x < 0, \end{array}\right. } \end{aligned}$$
  with $$\beta = 0.2$$.

A small slope $\beta$ = 0.2 was chosen for the LReLU to allow for a nonvanishing gradient over the domain of the function [39]. The output layer has a linear activation function. The DNN is implemented using the Keras package [40] with TensorFlow backend [41]. Back-propagation is done using stochastic gradient descent with the Adam optimizer [42].

### Hyperparameter Optimization

To optimize the performance of the DNN, three hyperparameters are considered: the depth of the network architecture, the dropout rate, and the gradient descent learning rate. They were tuned using the cross-validation algorithm [43]. The mean validation loss was used as the figure of merit for the optimization over a five-fold splitting of the training sample. The network has been trained on a single NVIDIA GeForce GTX 1080 Ti GPU.

Random sampling was used to select 50 of 120 grid points in hyperparameter space, where the grid is defined by the following:

- dropout rate: $$do \in [0.1, 0.2, 0.3, 0.4]$$.
- learning rate: $$lr \in [10^{-2}, 10^{-3}, 10^{-4}, 10^{-5}, 10^{-6}]$$.
- number of hidden layers: varied between 3 and 8.

The number of nodes in the last three hidden layers of the DNN was set to [512, 256, 128], respectively, while the number of nodes of the remaining layers was set to 1024. A number of configurations were found to provide comparable performance. Of these, the network with the smallest number of trainable parameters was chosen. The parameters and their values are: $do = 0.1$, $lr = 0.001$, and 6 hidden layers with [1024, 1024, 1024, 512, 256, 128] nodes. This architecture has a total of about 2.8 million trainable parameters.

### Training Set $p_{\mathrm {T}}$ Composition

The number of events as a function of the b jet $p_{\mathrm {T}}$ spectrum in the training sample spans six orders of magnitude, as shown in Fig. 1 (upper). This means that, during the training, the DNN is exposed to many more jets with low $p_{\mathrm {T}}$. In situations like this, one might expect worse performance for high-$p_{\mathrm {T}}$ jets. To check if this is an issue, emphasis was given to the high $p_{\mathrm {T}}$ part of the sample. About 95% of the jets with $p_{\mathrm {T}}$ below 400 $\,\text {GeV}$ were removed to reproduce the same exponential shape observed above 400 $\,\text {GeV}$. We found that the DNN trained on this subsample of events showed no improvement for high $p_{\mathrm {T}}$ jets, but did have up to 0.5% degradation of the relative jet energy resolution. For this reason, the final DNN is trained on the full sample.

## Results

The performance of the b jet regression was evaluated by comparing the b jet energy resolution and scale (defined as the most probable value of the $p_{\mathrm {T}} ^{\text {gen}}/ p_{\mathrm {T}} ^\text {reco}$ distribution), before and after the energy correction, on a test sample that is statistically independent from those used for training and validation. Different physics processes were included in the test set to evaluate the performance of the algorithm on b jets with different kinematics. The processes employed in the test sample are:

- $\hbox {t}\bar{\hbox {t}}$: top quark–antiquark pair production (independent of the training data set),
- $\hbox {Z}{}{} (\rightarrow \ell ^+\ell ^-)\hbox {H}{}{} (\rightarrow \hbox {b}\bar{\hbox {b}})$: associated production of a Higgs boson with a Z boson, where the Z boson decays to a pair of same flavor, opposite-charge electrons or muons, and the Higgs boson decays to $\hbox {b}\bar{\hbox {b}}$,
- $\hbox {H}{}{} (\rightarrow \hbox {b}\bar{\hbox {b}})\hbox {H}{}{} (\rightarrow {\upgamma }{}{} {\upgamma }{}{})$: double Higgs boson produced via gluon fusion with one Higgs boson decaying to $\hbox {b}\bar{\hbox {b}}$, and the other to a pair of photons, assuming both standard model (SM) and beyond SM kinematics. In the latter case, the double Higgs signal originates from the decay of a spin-0 resonance with a mass of 500 or 700 $\text {GeV}$.

**Fig. 2:**
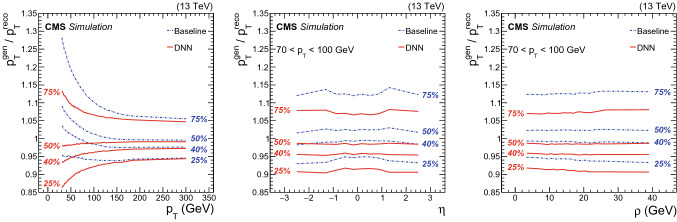
The 25, 40, 50, and 75% quantiles are shown for the b jet energy scale $p_{\mathrm {T}} ^{\text {gen}}/ p_{\mathrm {T}} ^\text {reco}$ distribution before (blue dashdot) and after (red solid) applying the regression correction as a function of jet $p_{\mathrm {T}}$ (left), $\eta$ (center), and $\rho$ (right). The $\eta$ and $\rho$ distributions are shown for jets with $p_{\mathrm {T}}$ $\in$ [70, 100] $\,\text {GeV}$

Figure 2 shows the 25, 40, 50, and 75% quantiles of the target distribution before and after applying the DNN b jet energy corrections, as a function of jet $p_{\mathrm {T}}$, $\eta$, and $\rho$. The results are obtained for b jets from the $\hbox {t}\bar{\hbox {t}}$test sample. The 40% quantile has been found to be a good approximation of the most probable value of the target distribution. In addition, the 40% quantile validates the performance on a quantile not used in the training. It can be seen that after DNN corrections, the distribution becomes narrower, and its median and 40% quantile exhibit smaller dependence on jet $p_{\mathrm {T}}$, $\eta$, and the median event energy density $\rho$.

**Fig. 3:**
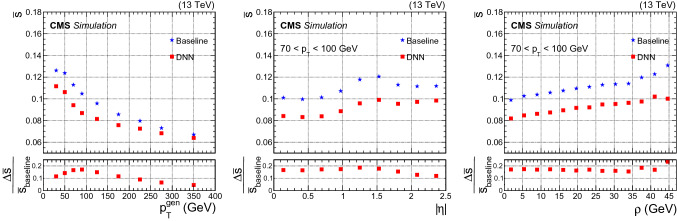
Relative jet energy resolution, $\overline{\mathrm {s}}$, as a function of generator-level jet $p_{\mathrm {T}} ^{\text {gen}}$ (left), $\eta$ (center), and $\rho$ (right) for b jets from $\hbox {t}\bar{\hbox {t}}$ MC events. The average $p_{\mathrm {T}}$ of these b jets is 80 $\text {GeV}$. The $\eta$ and $\rho$ distributions are shown for jets with $p_{\mathrm {T}}$ $\in$ [70, 100] $\text {GeV}$. The blue stars and red squares represent $\overline{\mathrm {s}}$ before and after the DNN correction, respectively. The relative difference $\varDelta \overline{\mathrm {s}}/\overline{\mathrm {s}} _{\text {baseline}}$ between the $\overline{\mathrm {s}}$ values before and after DNN corrections is shown in the lower panels

The jet energy resolution, $\mathrm {s}$, is estimated as half the difference between the 75% ($q_{75}$) and 25% ($q_{25}$) quantiles of the target distribution. To quantify the resolution improvement, we compared the relative jet energy resolution, $\overline{\mathrm {s}}$, defined as:

6 $$\begin{aligned} \overline{\mathrm {s}} \equiv \frac{\mathrm {s}}{q_{40}} = \frac{q_{75} - q_{25}}{2}\frac{1}{q_{40}}, \end{aligned}$$

where the resolution $\mathrm {s}$ is divided by $q_{40}$, the most probable value estimated as the 40% quantile of the target distribution. The relative improvement on $\overline{\mathrm {s}}$ for b jets for various physics processes is between 12 and 15%, as can be seen from Table 1. Figure 3 shows the value of $\overline{\mathrm {s}}$ obtained for b jets from the $\hbox {t}\bar{\hbox {t}}$ test sample as a function of the generator-level $p_{\mathrm {T}} ^{\text {gen}}$ (left), $\eta$ (center), and $\rho$ (right). The lower panels in Fig. 3 show the relative improvements resulting from the DNN energy correction. The observed behavior agrees with the expectation that the regression correction should optimize the jet energy resolution, while the baseline corrections aim for a flat response as a function of the jet generator level $p_{\mathrm {T}} ^{\text {gen}}$ and $\eta$. For all physics processes considered, the per-jet relative resolution improvement is around 12–18% for $p_{\mathrm {T}} <100\,\text {GeV}$, falling to around 5–9% for $p_{\mathrm {T}} >200\,\text {GeV}$. This improvement translates into an improvement in sensitivity of the analyses that make use of b jets in the final state. The improvement in the b jet energy resolution brought by the regression is similar for b jets with and without associated leptons. This demonstrates that the algorithm is able to correct not only for the undetected neutrinos in semileptonic decays of b hadrons, but also for effects that may only be present in hadronic decays. In addition, the regression was shown to improve the response of light jets by about 3%.

**Table 1:** Relative differences $\varDelta \overline{\mathrm {s}}/\overline{\mathrm {s}} _\text {baseline}$ between the $\overline{\mathrm {s}}$ values obtained before and after applying the DNN energy correction for b jets produced in the different physics processes indicated

MC sample	Improvement
$\hbox {t}\bar{\hbox {t}}$	12.2%
$\hbox {Z}{}{} (\rightarrow \ell ^+\ell ^-)\hbox {H}{}{} (\rightarrow \hbox {b}\bar{\hbox {b}})$	12.8%
$\hbox {H}{}{} (\rightarrow \hbox {b}\bar{\hbox {b}})\hbox {H}{}{} (\rightarrow {\upgamma }{}{} {\upgamma }{}{})$ SM	13.1%
$\hbox {H}{}{} (\rightarrow \hbox {b}\bar{\hbox {b}})\hbox {H}{}{} (\rightarrow {\upgamma }{}{} {\upgamma }{}{})$ resonant 500 $\text {GeV}$	14.5%
$\hbox {H}{}{} (\rightarrow \hbox {b}\bar{\hbox {b}})\hbox {H}{}{} (\rightarrow {\upgamma }{}{} {\upgamma }{}{})$ resonant 700 $\text {GeV}$	13.1%

Knowledge of jet energy resolution on a jet-by-jet basis can be exploited in analyses searching for resonant production of b jet pairs to increase their sensitivity. We have checked the correlation between the jet resolution $\mathrm {s}$ and the value of the per-jet resolution estimator, $\hat{\mathrm {s}}$, provided by the DNN:

7 $$\begin{aligned} \hat{\mathrm {s}} \equiv \frac{1}{2}({\hat{y}}_{75\%} - {\hat{y}}_{25\%}). \end{aligned}$$

**Fig. 4:**
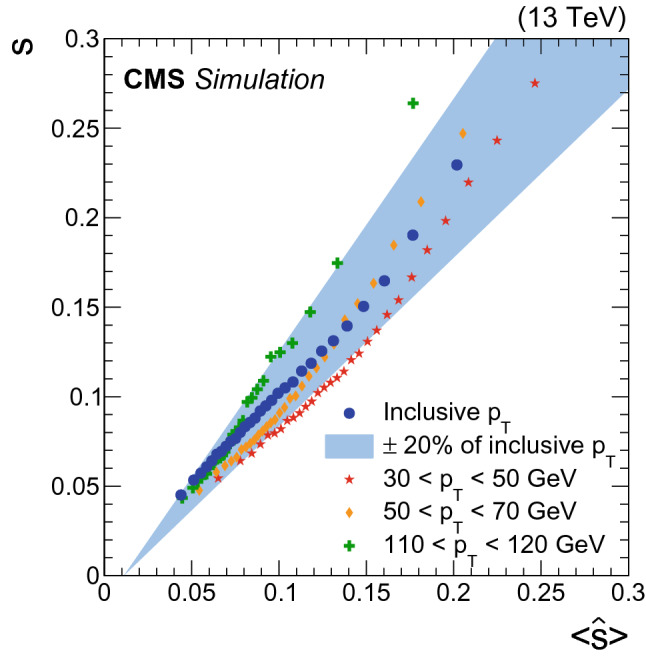
Correlation between jet energy resolution $\mathrm {s}$ and the average jet energy resolution estimator $\langle \hat{\mathrm {s}}\rangle$ for b jets from $\hbox {t}\bar{\hbox {t}}$ MC events. The blue circles correspond to the inclusive $p_{\mathrm {T}}$ spectrum, while the blue band represents 20% up and down variations of the fitted $\langle \hat{\mathrm {s}}\rangle$ trend for the inclusive $p_{\mathrm {T}}$ spectrum. The red stars correspond to jets with $p_{\mathrm {T}}$ $\in$ [30, 50] $\,\text {GeV}$, orange diamonds to $p_{\mathrm {T}}$ $\in$ [50, 70] $\,\text {GeV}$, and green crosses to $p_{\mathrm {T}}$ $\in$ [110,120] $\,\text {GeV}$

To do this, the sample of b jets was split into several equally populated bins in $\hat{\mathrm {s}}$. In each bin, the value of $\mathrm {s}$ is computed as half the difference between the $q_{75}$ and $q_{25}$ quantiles of the target distribution, and compared to the average resolution estimator $\langle \hat{\mathrm {s}}\rangle$. Figure 4 shows the correlation between $\mathrm {s}$ and the $\langle \hat{\mathrm {s}}\rangle$ values for the inclusive $p_{\mathrm {T}}$ spectrum and for several bins in $p_{\mathrm {T}}$. A linear dependence with slope near unity confirms that the per-jet energy resolution estimator $\hat{\mathrm {s}}$ correctly represents the jet resolution. We observe that deviations of the slope from unity from the linear behavior are roughly compatible within 20% of the $\hat{\mathrm {s}}$ value.

**Fig. 5:**
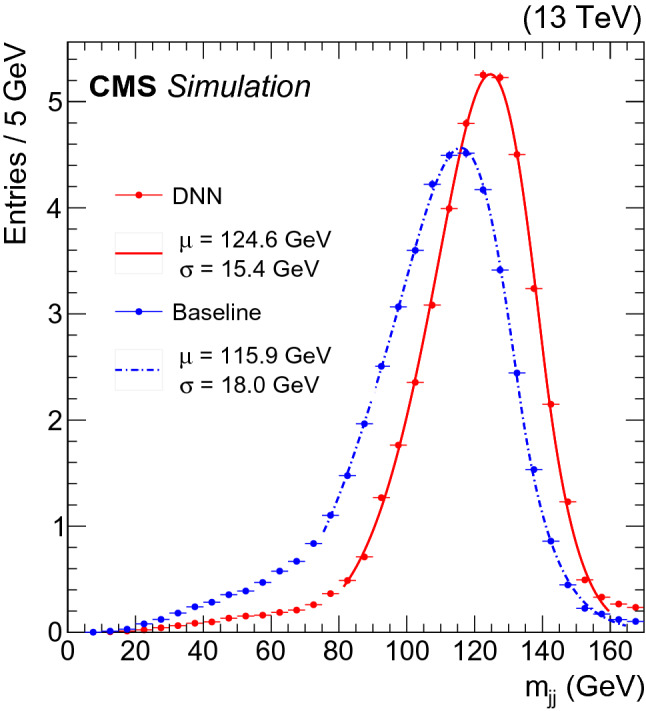
Dijet invariant mass distributions for simulated samples of $\hbox {Z}{}{} (\rightarrow \ell ^+\ell ^-)\hbox {H}{}{} (\rightarrow \hbox {b}\bar{\hbox {b}})$ events, where two jets and two leptons were selected. Distributions are shown before (dotted blue) and after (solid red) applying the b jet energy corrections. A Bukin function [44] was used to fit the distribution. The fitted mean and width of the core of each distribution are displayed in the figure

While the improvements described above are quoted at the single jet level, many physics analyses use the invariant mass of the two b jet system as a discriminating variable for signal extraction. The improvement in the resolution of the dijet invariant mass is generally bigger than that for a single jet, because the energy corrections effectively equalize the energy scale of the two jets, while also improving the jet resolution. To estimate the dijet resolution, improvement, events with two leptons and two jets were selected from the $\hbox {Z}{}{} (\rightarrow \ell ^+\ell ^-)\hbox {H}{}{} (\rightarrow \hbox {b}\bar{\hbox {b}})$ sample: jets were required to have $p_{\mathrm {T}}$ larger than 20 $\text {GeV}$, absolute value of $\eta$ below 2.4, and be compatible with the hadronisation of b quarks, referred to as “b-tagged” [37] jets in the following. The selection criteria for the b-tagged jets correspond to a 70% b jet tagging efficiency with a 1% misidentification rate for light-flavor or gluon jets. Leptons were required to have a $p_{\mathrm {T}}$ larger than 20 $\text {GeV}$, while the lepton pairs were required to be compatible with the decay of a Z boson, requiring their invariant mass to be within 20 $\text {GeV}$ of the mass of the Z boson. The Z boson was required to have a transverse momentum larger than 150 $\text {GeV}$. An improvement of about 20% in the dijet invariant mass resolution in the $\hbox {Z}{}{} (\rightarrow \ell ^+\ell ^-)\hbox {H}{}{} (\rightarrow \hbox {b}\bar{\hbox {b}})$ sample can be observed in Fig. 5. A Bukin function [44] was used to fit the core of the distribution in Fig. 5. The fit is performed in the range [75, 165] $\text {GeV}$ for the baseline and [81,160] $\text {GeV}$ for the DNN corrected distribution.

In addition, a dedicated study was performed to test how well the algorithm performance can be transferred from Monte Carlo simulations to the domain of pp collision data. A set of Z boson candidates decaying to a pair of charged leptons was extracted from pp collisions recorded by the CMS experiment in 2017. A standard set of requirements [28, 45] was applied to select events with electron or muon pairs compatible with having originated from the decay of a Z boson. Events were further required to have at least one b-tagged jet. The jet with the largest $p_{\mathrm {T}}$ was required to have $|\eta | < 2$, while the $p_{\mathrm {T}}$ of the dilepton system was required to be larger than 100 $\text {GeV}$. The $p_{\mathrm {T}}$ balance between the Z boson and the b-tagged jet candidate was enforced by requiring that extra jets have a $p_{\mathrm {T}}$ less than 30% of the Z $p_{\mathrm {T}}$ to suppress events with additional hadronic activity. Events satisfying these requirements were used to evaluate the agreement between data and MC simulations. In addition, the resolution of the jets was measured by extrapolating to zero additional hadronic activity following the methodology described in Ref. [28].

**Fig. 6:**
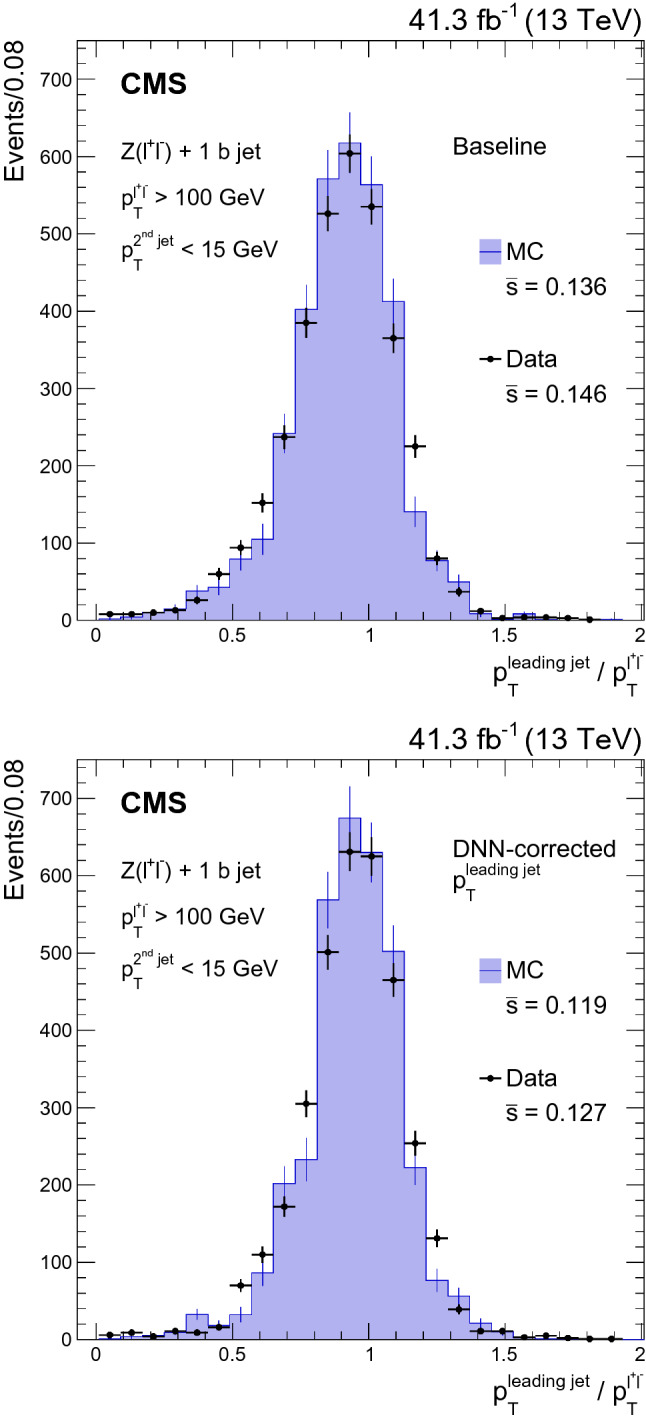
Distribution of the ratio between the transverse momentum of the leading b-tagged jet and that of the dilepton system from the decay of the Z boson. Distributions are shown before (upper) and after (lower) applying the b jet energy corrections. The $\overline{\mathrm {s}}$ values of the core distributions are included in the figures. The black points and histogram show the distributions for data and simulated events, respectively.

Figure 6 shows the ratio between the $p_{\mathrm {T}}$ of the leading jet and that of the dilepton system for events in which the $p_{\mathrm {T}}$ of the subleading jet is less than 15 $\text {GeV}$. The upper and lower panels show the distributions obtained before and after applying the DNN-based corrections, respectively. It can be seen that the effect of the corrections is to reduce the width of the distribution. Using the method detailed in Ref. [28], the double ratio of the relative jet resolution $\overline{\mathrm {s}}$ measured in data and in simulated events was found to be $1.1 \pm 0.1$ before and after applying the DNN-based corrections. This validates that the resolution improvement achieved in simulated events is successfully transferred to the data domain.

## Summary

We have described an algorithm that makes it possible to obtain point and dispersion estimates of the energy of jets arising from b quarks in proton–proton collisions. We trained a deep, feed-forward neural network, with inputs based on jet composition and shape information, and on properties of the associated reconstructed secondary vertex for a sample of simulated b jets arising from the decays of top quark–antiquark pairs. The neural network simultaneously finds robust mean, 25 and 75% quantile estimators for the energy of a b jet. The mean estimator is based on the Huber loss function and is used as an energy correction, while the 25 and 75% quantile estimators are used to build a jet-by-jet resolution estimator, defined as half the difference between these quantiles.

The DNN-based algorithm leverages the information contained in a large training data set consisting of nearly 100 million simulated b jets, and improves the resolution of the b jet energy by 12–15% relative to that which is found after baseline corrections. An improvement of about 20% is observed in the resolution of the invariant mass of b jet pairs resulting from the decay of a Higgs boson produced in association with a Z boson. The resolution estimator is further shown to predict the resolution of b jets with an accuracy of 20% over a $p_{\mathrm {T}}$ range between 30 and 350 $\text {GeV}$. Events containing a dilepton decay of a Z boson produced in association with a b jet are used to validate the performance of the algorithm on proton–proton collision data recorded with the CMS detector. The jet energy resolution improvement observed in data is consistent with that found in simulation.

The results described here are being used by the CMS Collaboration in several physics analyses targeting the final states containing b jets, including the observation of the Higgs boson decay to $\hbox {b}\bar{\hbox {b}}$ [13].
